# Intradermal immunization of mice with radiation-attenuated sporozoites of *Plasmodium yoelii *induces effective protective immunity

**DOI:** 10.1186/1475-2875-9-362

**Published:** 2010-12-15

**Authors:** Tatiana Voza, Chahnaz Kebaier, Jerome P Vanderberg

**Affiliations:** 1 Department of Medical Parasitology, New York University School of Medicine, 341 East 25th Street, New York, NY 10010, USA; 2 Biological Sciences Department, The City University of New York, 300 Jay Street, Brooklyn, NY 11201, USA; 3 Department of Medicine, Division of Infectious Diseases, University of North Carolina, Chapel Hill NC, 27599-7030, USA

## Abstract

**Background:**

Intravenous injection of mice with attenuated *Plasmodium berghei *sporozoites induces sterile immunity to challenge with viable sporozoites. Non-intravenous routes have been reported to yield poor immunity. Because intravenous immunization has been considered to be unacceptable for large scale vaccination of humans, assessment was made of the results of intradermal immunization of mice with *Plasmodium yoelii*, a rodent malaria parasite whose infectivity resembles that of human malaria.

**Methods:**

Mice were immunized with two injections of isolated, radiation-attenuated *P. yoelii *sporozoites, either by intravenous (IV) or intradermal (ID) inoculation. In an attempt to enhance protective immunogenicity of ID-injections, one group of experimental mice received topical application of an adjuvant, Imiquimod, while another group had their injections accompanied by local "tape-stripping" of the skin, a procedure known to disrupt the stratum corneum and activate local immunocytes. Challenge of immunized and non-immunized control mice was by bite of sporozoite-infected mosquitoes. Degree of protection among the various groups of mice was determined by microscopic examination of stained blood smears. Statistical significance of protection was determined by a one-way ANOVA followed by Tukey's *post hoc *test.

**Results:**

Two intravenous immunizations produced 94% protection to mosquito bite challenge; intradermal immunization produced 78% protection, while intradermal immunization accompanied by "tape-stripping" produced 94% protection. There were no statistically significant differences in degree of protective immunity between immunizations done by intravenous versus intradermal injection.

**Conclusions:**

The use of a sub-microlitre syringe for intradermal injections yielded excellent protective immunity. ID-immunization with large numbers of radiation-attenuated *P. yoelii *sporozoites led to levels of protective immunity comparable to those achieved by IV-immunization. It remains to be determined whether an adjuvant treatment can be found to substantially reduce the numbers of attenuated sporozoites required to achieve a strong protective immunity with as few doses as possible for possible extension to immunization of humans.

## Background

In spite of the huge global morbidity and mortality inflicted by malaria, an effective and practical vaccine against this disease has not yet been achieved. Early studies on immunization against sporozoite-induced rodent malaria resulted in close to 100% protection when mice were immunized with *Plasmodium berghei *sporozoites irradiated to sufficient levels and the immunized mice were subsequently challenged with non-irradiated sporozoites [1,2].

Nevertheless, immunization with attenuated *P. berghei *sporozoites via routes other than intravenous (IV) was found to be far less protective. Thus, even after five immunizations, mice immunized by intramuscular (IM), intraperitoneal (IP) or intradermal (ID) routes were protected only 32%, 26% and 24%, respectively, in contrast to 95% protection after IV immunization [3]. In another *P. berghei *study done with similar protocols, IM immunization resulted in only 11% protection, although addition of albumin to the immunizing inoculum raised this to 42%; in contrast, IV immunization yielded 100% protection [4].

Because sporozoite suspensions used for immunization were heavily contaminated with microorganisms and mosquito components, it was clear that such immunization trials by IV injection could not be directly extended to humans. An alternate approach, however, allowed irradiated mosquitoes to directly inoculate attenuated sporozoites into hosts, the mosquitoes thereby acting as vehicles of immunization. This approach was first established with rodent malaria [5] and then extended to the first successful human vaccination trial against *P falciparum *malaria [6]. A compendium of subsequent human vaccination trials with this approach showed that when sufficient numbers of mosquitoes were used for immunization, greater than 90% of volunteers were completely protected against challenge by bite of infected mosquitoes [7,8]. Recent progress by this group, under the auspices of the biopharmaceutical company Sanaria, has permitted the raising of large numbers of mosquitoes infected with *Plasmodium falciparum *sporozoites, the purification of these sporozoites sufficient to render them acceptable for human vaccination, and the successful freeze-preservation of the attenuated sporozoites. Trials are currently underway to attempt to vaccinate humans by syringe injection of these sporozoites [9,10].

A central question for any human trials relates to an appropriate route of immunization. It had long been assumed that most sporozoites injected by mosquitoes rapidly reach the blood, after which they travel to the liver for further development. Thus, there was a supposition that sporozoite inoculation by mosquitoes mimicked IV inoculation of sporozoites by syringe. But studies have shown that most if not all mosquito-injected sporozoites are deposited into avascular portions of the skin and sub-cutaneous tissues and that sporozoites then use gliding motility to reach blood vessels to travel to the liver [11,12], or enter lymph vessels to travel to local draining lymph nodes [12]. This has led to the possibility that inoculation of isolated sporozoites directly into the skin by syringe might successfully replicate the recognized successful approach of allowing mosquitoes to inoculate attenuated sporozoites into skin.

Accordingly, the *Plasmodium yoelii *rodent malaria system was used to explore this approach and investigate ways of further enhancing the protective immunogenicity of ID-injected, attenuated sporozoites. The *P. yoelii *system is far superior to the *P. berghei *system in the infectivity of sporozoites, appearing to be similar in that respect to the human malarias. Indeed, others have contended that "the *P. yoelii *system has accurately predicted the success or failure of every approach to malaria vaccination that has been tested in humans" [13]. A recent publication reported that it took at least four ID immunizations with a total of 6,000 *P. yoelii *sporozoites to equal the protective immunity that could be accomplished with only three doses and a total of only 2,250 sporozoites administered IV [14]. Because of the practical value of achieving protective immunity with fewer doses of sporozoites, the current study tested whether a substantially higher number of sporozoites given in only two doses ID might result in protection equivalent to that obtained with IV immunization.

The main goal was to assess the protective immunogenicity of attenuated *P. yoelii *sporozoites injected by syringe into the skin. ID immunization with *P. yoelii *sporozoites was found to give far better protection than had been observed in previous attempts using non-IV routes of administration to immunize with *P. berghei *sporozoites [3,4]. Furthermore, administering larger numbers of *P. yoelii *sporozoites ID was able to give a degree of protective immunity equivalent to what had been reported for ID immunization with twice the number of immunizing doses [14].

Finally, because many sporozoites injected by mosquitoes remain in the skin and either deteriorate [11] or differentiate [15] and because such sporozoites may be involved in induction of the immune response [16], two methods were tested to possibly enhance the immune response with adjuvants in conjunction with ID-administration of sporozoites; a) topical application of the toll-like receptor (TLR) agonist Imiquimod, as previously done for immunization studies using sub-unit vaccines against malaria [17], b) "tape-stripping" (TS), a procedure known to disrupt the stratum corneum and enhance the immunogenicity of ID-injected antigens [18]. All ID-injection procedures were found to lead to levels of protection not significantly different from that which had been observed after IV immunization.

## Methods

### Host-parasite system

*Plasmodium yoelii *sporozoites (strain 17XNL) were produced in *Anopheles stephensi *mosquitoes. Standard protocols were used for infecting and maintaining mosquitoes [19], which were infected by feeding upon gametocyte-carrying 6-8 wk-old Swiss-Webster mice (Taconic Farms Inc., Germantown, NY). Protocols for maintenance and use of experimental animals were approved by the Institutional Animal Care and Use Committee at New York University School of Medicine, whose animal facility is accredited by the Association for Assessment and Accreditation of Laboratory Animal Care International (Rockville, MD).

### Isolation of sporozoites for immunization

Sporozoites for immunization were isolated from mosquitoes 18 days after the mosquitoes had received an infective blood meal. To obtain sporozoites, mosquitoes were anesthetized on ice, then washed with 70% ethanol, followed by RPMI medium 1640 (Gibco BRL, Grand Island, NY). Salivary glands were dissected out and triturated in RPMI medium supplemented with 2% mouse serum albumin, after which the freed sporozoites were counted in a haemacytometer and diluted to appropriate concentrations for immunization.

### Immunization and challenge protocols

Sporozoites were irradiated within a gamma irradiator (MDS Nordion Gammacell® 1000 Elite) to a central dose of ± 12,049 cGy and a minimum dose of ± 10,266 cGy. Mice (BALB/c females from Taconic Farms Inc., Germantown, NY) were 6 wk old at the initiation of each experiment (n = 6/group for each experiment). For immunization, they received an initial injection of 60,000 irradiated sporozoites, with a subsequent booster injection of 30,000 irradiated sporozoites 15 days afterwards. Intravenous (IV) injections into a tail vein were given in a volume of 200 μl per mouse. For intradermal (ID) immunizations, a portion of flank skin was shaved and injections were given with a Nanofil TM Sub-Microliter syringe with a 33G flexifil beveled tip (World Precision Instruments, Sarasota, Florida, USA) in two adjacent sites in a volume of 5 μl per site.

Parallel ID immunizations were given to groups of mice that received topical treatment with an adjuvant or an adjuvant procedure. For topical treatment with Imiquimod (obtained as a 5% cream containing 12.5 mg/ml of Imiquimod [Aldara; 3 M, St. Paul, MN]), anesthetized mice were treated with approximately 25 μl (1.25 mg of imiquimod) [17]. It was applied by rubbing into the shaved skin area 4 h after ID immunization, then reapplied 1 and 2 days after immunization. This process was repeated for the booster immunization.

For assessment of the adjuvant effect of TS, a small area of the shaved flank skin of anesthetized mice was treated by applying and pulling off a strip of adhesive tape (Scotch Brand 3 M Magic tape) 10 times. A fresh piece of tape was used for each of the 10 strippings. This procedure is known to disrupt the stratum corneum [18], and resulted in local erythema and inflammation typical of what has been demonstrated in previous studies with tape-stripped mice [18]. Tape-stripping was done 30 min after ID immunization with sporozoites and was repeated for the booster immunization.

Mice were anesthetized and then challenged by mosquito bite 15 days after the second immunization. Parallel challenges were done on non-immunized control mice from the same cohort. For each mouse, five different infected mosquitoes from the same cohort were allowed to probe and feed for 5 min. Thin blood smears were prepared from drops of tail blood up to 14 days after challenge and stained with Giemsa.

### Statistics

The percentages of mice that developed parasitemia and the pre-patent periods of those that developed parasitemia after challenge by mosquito bite were recorded. Comparisons were made between the percentages of mice infected after the various immunization regimens (IV, ID, ID+imiquimod, ID + TS) versus the percentage of non-immunized controls that were infected. To do these comparisons, all data were transformed using the following equation y = arcsin[√(y/100)], where y represents percentage of infection. Gaussian distribution of transformed data was then confirmed using the Kolmogorov-Smirnov normality test. A one-way ANOVA followed by Tukey's *post hoc *test was then used to compare the differences between groups of mice. The analyses were performed using GraphPad Prism Version 5 software (San Diego, California).

## Results

### Challenges by mosquito injection of sporozoites

The results of three independent experiments are shown in Table 1. Mice immunized by two IV injections of sporozoites and challenged by mosquito bite had an overall protection of 94%, compared with non-immunized controls. Mice immunized by ID injections had an overall protection of 78%. When attempts were made to enhance ID immunizations by adjuvant treatments, treatment with Imiquimod resulted in 67% protection, while treatment by TS gave 94% protection. In spite of the fact that the percentage of mice protected after ID immunization with TS was the same as the percentage protected after IV immunization, it was not possible to demonstrate that these were significantly better than protection observed after ID immunization alone (P > 0.05).

**Table 1:** Effect of immunization protocols with Plasmodium yoelii attenuated sporozoites on development of protective immunity

Route of Immunization	Exp't 1	Exp't 2	Exp't 3	Total
Non-Immunized Controls	6/6 (0%)^a^ [PP = 3.0]^b^	6/6 (0%) [PP = 3.3]	6/6 (0%) [PP = 3.3]	18/18 (0%) [PP = 3.3]
IV^c^-Immunized	0/6 (100%)	0/6 (100%)	1/6 (83%) [PP = 6.0]	1/18 (94%) [PP = 6.0]
ID^d^-Immunized	3/6 (50%) [PP = 5.0]	1/6 (83%) [PP = 9.0]	0/6 (100%)	4/18 (78%) [PP = 6.0]
ID-Immunized + Imiquimod	1/6 (83%) [PP = 5.0]	1/6 (83%) [PP = 4.0]	4/6 (33%) [PP = 4.2]	6/18 (67%) [PP = 4.3]
ID-Immunized + Tape Stripping	1/6 (83%) [PP = 4 0]	0/6 (100%)	0/6 (100%)	1/18 (94%) [PP = 4.0]

^a ^Results of challenge by mosquito injection of sporozoites. # of mice with blood infection/# challenged; (% protected in parentheses)

^b ^[Mean prepatent period in days for positive mice]

^c ^IV = intravenous

^d ^ID = intradermal

## Discussion

A proof of concept study done more than 35 years ago showed the possibility of immunizing humans against malaria by irradiating infected mosquitoes and allowing them to inject attenuated *P. falciparum *sporozoites into volunteers [6]. In subsequent human trials with sporozoite vaccination via mosquito injection, greater than 90% of volunteers were protected against challenge by bite of irradiated, infected mosquitoes [7,8]. Nevertheless, the impracticality of immunization by mosquito bite was obvious. A more recently proposed approach is based on syringe injection of purified and freeze-preserved, attenuated *P. falciparum *sporozoites isolated from mosquito salivary glands [9,10].

However, early studies with *P. berghei *sporozoites suggested that immunization by syringe injection of sporozoite suspensions was not very effective unless the sporozoite suspensions were administered IV. As this is not a feasible mode of administration for large scale immunization of humans, more suitable modes of syringe injection of sporozoites were revisited with another species of rodent malaria, *P. yoelii*. The rationale for this is that far fewer *P. yoelii *than *P. berghei *sporozoites are required, either to infect [20,21] or to protectively immunize [22] mice.

Results for three experiments in the present study showed an overall 94% protection for mice immunized IV vs. 78% for ID immunization after only two immunizations. An initial pilot experiment using three immunizations for this study had yielded 100% protection after IV immunization, compared to 83% protection with ID immunization (data not shown). It has been reported that two IV doses of irradiated *P. yoelii *sporozoites rarely provides solid protection, while three IV doses does so consistently [22,23]. Thus, to better discriminate between the effects of various types of immunization protocols with and without adjuvant treatments, subsequent immunization experiments were performed with only two immunizations, as presented in this paper. It was anticipated that such suboptimal immunization might better differentiate the effects of adjuvant treatments. Furthermore, it has been noted that a goal for eventual human vaccination is to achieve high-level protection with the fewest numbers of doses of vaccine [10].

When adjuvant treatments in conjunction with the ID immunizations were evaluated, it was found that topical application of Imiquimod resulted in an overall protection rate of 67%, whereas TS gave 94% protection. IV immunization is the generally accepted "gold standard" for protection achieved with attenuated sporozoites. Although TS in conjunction with ID immunization yielded the same degree of protection as IV-immunization, it was not possible to show statistically that the protection induced by ID immunization together with TS was significantly better than ID immunization alone. Imiquimod is a TLR agonist that stimulates local immunocytes, including dermal dendritic cells, when topically applied [24]. It has been successfully used as an adjuvant to increase protective immunity against *Leishmania major *in BALB/c mice [25] and to enhance immunogenicity of a peptide-based vaccine against *Plasmodium *sporozoites [17]. However, topically applied Imiquimod showed no enhancing effects when used in association with ID-injection of radiation attenuated sporozoites.

Tape stripping together with ID-injection of sporozoites, on the other hand, protected the same percentage of mice as observed after IV immunization. Tape stripping consists of partially removing the stratum corneum, the outermost layer of the epidermis, by use of adhesive strips applied to the skin and then removed [18]. This procedure had been shown to enhance the immune response to an ID-injected protein antigen in the tape-stripped area [18] and [26]. It is known to result in local inflammation, with movement of antigen-presenting cells into affected regions and enhancement of immune surveillance [26], as well as induction of TLR-9 mRNA [27]. The rationale for using TS was based on previous observations that mosquitoes inject sporozoites into avascular portions of skin and subcutaneous tissues and that many sporozoites remain in the skin and become fragmented within several hours [11] and [28]. Furthermore, it has been shown that an anti-sporozoite response originates early in lymphoid tissues linked to cutaneous infection sites and it was suggested that this originates with internalization of sporozoite antigen by immature dendritic cells in the skin [16]. The current study hypothesized that the effects of TS might enhance interactions between immunocytes and sporozoites deposited in the skin.

The 78% protection reported here after simple ID administration of radiation-attenuated *P. yoelii *sporozoites with only two immunizations is substantially higher than any non-IV route of immunization previously reported with *P. berghei *sporozoites. A prior study by others had also reported good protective results after non-IV immunization with attenuated *P. yoelii *sporozoites [14]. Nevertheless, that study had reported that four doses of *P. yoelii *sporozoites given ID or SC were required to achieve protection similar to what was achieved with three doses of fewer sporozoites administered IV. Those results, as well as the ones reported here, are encouraging because it has been argued that "experiments using attenuated *P. yoelii *sporozoite vaccines have consistently paralleled or predicted results in humans exposed to *P. falciparum *sporozoites" [22]. Because of the practical operational value of achieving protective immunity with as few doses of sporozoites as possible, the present study tested whether only two ID doses given with a relatively high number of sporozoites might result in protection comparable to that obtained with IV immunization. The current study now shows that this is the case with *P. yoelii *malaria. When two immunizing doses with large numbers of sporozoites were used, there were no significant differences between results obtained with IV-versus ID-immunization. Indeed, ID-immunization accompanied by TS gave the same degree of protection obtained with the IV immunization "gold standard. These results are in accord with a retrospective analysis showing that immunization of humans with *P. falciparum *sporozoites injected by irradiated mosquitoes was most effective when large numbers of immunizing mosquito bites were used to introduce larger numbers of attenuated sporozoites into the vaccinees. Immunization with greater than 1,000 such bites led to total protection in 33 of 35 challenges, with protection lasting up to 10.5 months [9]. It remains to be determined whether an adjuvant treatment in conjunction with ID-immunization by syringe can be found to substantially reduce the numbers of attenuated sporozoites required to achieve a strong protective immunity with as few immunizing doses as possible.

## Conclusion

ID-immunization with large numbers of radiation-attenuated *P. yoelii *sporozoites with as few as two immunizing doses leads to levels of protective immunity comparable to those achieved by IV-immunization.

## Competing interests

The authors declare that they have no competing interests.

## Authors' contributions

All authors shared in experimental design, conduct of experiments, collection of data and experimental analysis. All authors helped to finalize the manuscript and approved the final manuscript.
